# Draft Genome Sequence of *Comamonas* sp. Strain E6 (NBRC 107749), a Degrader of Phthalate Isomers through the Protocatechuate 4,5-Cleavage Pathway

**DOI:** 10.1128/genomeA.00643-15

**Published:** 2015-06-18

**Authors:** Jun Shimodaira, Naofumi Kamimura, Akira Hosoyama, Atsushi Yamazoe, Nobuyuki Fujita, Eiji Masai

**Affiliations:** a Biological Resource Center, National Institute of Technology and Evaluation, Nishihara, Shibuya-ku, Tokyo, Japan; b Department of Bioengineering, Nagaoka University of Technology, Kamitomioka, Nagaoka, Niigata, Japan

## Abstract

*Comamonas* sp. strain E6 can degrade *o*-phthalate, terephthalate, and isophthalate via the protocatechuate 4,5-cleavage pathway. Here, we report the draft genome sequence of E6 in order to provide insights into its mechanisms in *o*-phthalate catabolism and its potential use for biotechnological applications.

## GENOME ANNOUNCEMENT

*Comamonas* sp. strain E6, isolated in Niigata, Japan, is a betaproteobacterial strain capable of degrading phthalate isomers, *o*-phthalate, terephthalate, and isophthalate (1), through the protocatechuate 4,5-cleavage (PCA45) pathway (2). In this strain, the functions and transcriptional regulations of *tph*, *iph*, and *pmd* genes responsible for the catabolism of terephthalate, isophthalate, and protocatechuate (PCA), respectively, have been characterized (1, 3–7). In addition, a novel tripartite aromatic acid transporter (TAAT) involved in the uptake of terephthalate and isophthalate was discovered (8). Since 2-pyrone-4,6-dicarboxylic acid (PDC), an intermediate from PCA via the PCA45 pathway, is an attractive chemical building block for the synthesis of biodegradable and highly functional polymers (9–13), elucidating further degradation mechanisms of phthalate isomers by strain E6 may provide new insight into an efficient production of PDC from inexpensive phthalate isomers. For this purpose, we decided to determine the whole-genome shotgun sequence of strain E6.

The genomic DNA of strain E6 was sequenced by a combined method of shotgun sequencing using 454 GS FLX Titanium (Roche, Basel, Switzerland) and paired-end sequencing using HiSeq 1000 (Illumina, San Diego, CA, USA). A hybrid assembly of the 454 GS FLX Titanium single-end data (210,535 reads, 16-fold coverage) and the Illumina paired-end data (5,483,902 reads, 91-fold coverage) was performed with Newbler version 2.6 (Roche). Contigs obtained from the assembly and sequences determined in previous studies (1, 4, 5, 8) were further assembled with Sequencher version 5.1 (Gene Codes Corp, Ann Arbor, MI, USA). The resulting contigs were analyzed using the Microbial Genome Annotation Pipeline (MiGAP) (http://www.migap.org/) for predicting protein-coding, tRNA, rRNA genes, and functional annotation of protein-coding genes. Functional annotations of the predicted protein-coding genes were refined using a protein BLAST search against the NCBI nonredundant database. The draft genome of strain E6 consists of 72 contigs (contig size range, 531 to 551,292 bp) with a total size of 5,576,776 bp, 107-fold sequencing coverage, *N*_50_ length of 186,733 bp, and 61.07% G+C content. The annotation revealed 5,131 predicted protein-coding sequences (CDSs), 3 rRNA genes, and 56 tRNA genes.

The *ophA1*, *ophA2*, *ophB*, and *ophC* genes (CSE6_002_00610, CSE6_002_00660, CSE6_002_00650, and CSE6_002_00630) responsible for *o*-phthalate degradation, encoding reductase component of phthalate 4,5-dioxygense, oxygenase component of phthalate 4,5-dioxygense, *cis*-phthalate dihydrodiol dehydrogenase, and 4,5-dihydroxyphthalate decarboxylase, respectively, were newly found upstream of the *tph* genes. In addition, a new gene (CSE6_002_00570) predicted as encoding a receptor protein of the TAAT system was also found downstream of the *oph* genes and may be involved in the uptake of *o*-phthalate.

The 16S rRNA gene sequence from strain E6 has 100% similarity with those of type strains *Comamonas thiooxydans* S23^T^ (DQ322069) and *Comamonas testosteroni* ATCC 11996^T^ (AHIL01000001). The analysis of average nucleotide identities (ANI) of strain E6 using the ANI calculator (http://enve-omics.ce.gatech.edu/ani/) showed 93.91% and 98.57% ANI values with *C. testosteroni* NBRC 14951^T^ (BBJZ00000000) and *C. thiooxydans* DSM 17888^T^ (BBVD00000000), respectively. The phylogenetic affiliation of strain E6 was closely related to *C. thiooxydans*.

### Nucleotide sequence accession numbers. 

The draft genome sequence of strain E6 has been deposited in the DDBJ/EMBL/GenBank databases under the accession no. BBXH00000000. The version used here is BBXH01000000.
